# Comparison of self-esteem and quality of life in 8-12-year-old children with ADHD with and without learning disorders

**DOI:** 10.1186/s40359-024-01732-7

**Published:** 2024-04-20

**Authors:** Armon Massoodi, Sussan Moudi, Maryam Malekiamiri, Hemmat Gholinia Ahangar

**Affiliations:** 1 Assistant Professor of child and adolescent Psychiatry, Social Determinants of Health Research Center, Health Research Institute, Babol University of Medical Sciences, Babol, IR Iran; 2 Associate Professor of Psychiatry, Social Determinants of Health Research Center, Health Research Institute, Babol University of Medical Sciences, Babol, Iran; 3 Student Research Committee, Babol University of Medical Sciences, Babol, Iran; 4 Clinical Research Development Unit of Rouhani Hospital, Babol University of Medical Sciences, Babol, Iran

**Keywords:** Self-esteem, Quality of life, Children, ADHD, Learning disorders

## Abstract

**Background:**

Attention Deficit/Hyperactivity Disorder (ADHD) is one of the most common disorders in school-aged children. Learning disorder (LD) is also one of the most important psychiatric disorders in children, which can often be associated with ADHD. In this study, we sought to compare self-esteem and quality of life in 8 to 12-year-old children with attention deficit/hyperactivity disorder with and without co-occurring learning disorders in order to emphasize the importance of attention and diagnosis in children with ADHD.

**Method:**

Among the 8- to 12-year-old outpatients referred to the child and adolescent psychiatry clinic of Omid Babol Clinic, 120 children aged 8 to 12 years with attention deficit/hyperactivity disorder whose disease was diagnosed by a child and adolescent psychiatry subspecialist. Among the tools used to collect information was the Colorado Learning Difficulties Questionnaire by Wilcott et al. (CLDQ), five-scale self-esteem test of Pepe et al. (1989) for children and quality-of-life questionnaire for 8–12-year-old children (PedsQL).

**Results:**

This study investigated self-esteem and quality of life in children with ADHD (*n* = 120, 51.7% boys). Children with ADHD and learning disabilities reported significantly lower self-esteem and quality of life compared to those with ADHD alone.

**Conclusion:**

Considering the relatively high probability of co-occurrence of ADHD and learning disorders, if one of them is diagnosed in a child, it is possible to look for other disorders in the child in order to avoid the more severe negative effects that this co-occurrence can have on the child by diagnosing it as soon as possible.

## Introduction

Attention Deficit/Hyperactivity Disorder (ADHD) is one of the most common disorders in school-aged children and is considered one of the main reasons for referral to psychiatrists and child psychologists. This disorder is characterized by a persistent pattern of inattention or impulsive and hyperactive behaviors. Affected children have a major weakness in executive functioning, which leads to many problems in planning and purposeful behavior at school, home, and communicating with others. Affected children are exposed to all kinds of injuries, including academic problems, behavioral disorders, and the risk of other disorders increasing. Therefore, in order to reduce the damage caused by the above problems, early intervention in the home and school environment becomes necessary [1, 2]. A review of the prevalence of ADHD shows that there is a significant percentage of school children with ADHD among relatively diverse populations (geographically, racially, socially, and economically). Although early prevalence estimates vary between 1% and 20%, the consensus of experts is that it is approximately 3–5% of the school-age population in the West and in India, the few studies that have assessed ADHD have a prevalence of They report between 5 and 10%. These significant changes in recorded prevalence rates do not result from actual differences in prevalence, but from important methodological differences between different studies such as changes in terms used, diagnostic methods, diagnostic systems and related criteria, differences in the number of information sources required for be diagnosed and sampled from the population [3]. Learning disorder (LD) is also one of the most important psychiatric disorders in children, which can often be associated with ADHD. be Children with learning disabilities are known to have academic problems. Perhaps for this reason, their other issues have received less attention. So that in addition to academic problems, this group also experiences other deficiencies that are sometimes considered by some theories as the basis of their learning and academic problems, such as fear of failure, depression, anxiety and loneliness [4, 5]. The prevalence of learning disorders in children varies depending on the specific disorder and the study population. According to a systematic review and meta-analysis, the overall prevalence of specific learning disabilities (SLD) in India was 8% (95% CI = 11 − 4) [6]. In a group of children with school failure, the prevalence of SLDs was 32%, which represents a general population prevalence of 6.4% [7].

Despite the high prevalence of learning disorders in children with ADHD, and the adverse consequences it has on the academic and social performance of these children, it seems that little attention has been paid to the accompanying and consequences of these disorders in children with ADHD. The relationship between self-esteem and quality of life in children with ADHD and learning disorders is not directly presented in the search results. However, the results discuss various aspects of health and well-being in children with ADHD and learning disorders, including quality of life, self-esteem, and the impact of comorbidities on their overall well-being [8–10]. So, in this study, we sought to compare self-esteem and quality of life in 8 to 12-year-old children with attention deficit/hyperactivity disorder with and without co-occurring learning disorders in order to emphasize the importance of attention and diagnosis in children with ADHD. Since no similar study has been conducted in Iran and also in the current study, confounding factors and assimilation of factors such as gender and age have been carefully considered as much as possible, from this point of view it can be considered important.

## Methods

### Sampling

The statistical population of this cross-sectional study includes all 8 to 12-year-old children with attention deficit/hyperactivity disorder with and without learning disorders who were referred to the child and adolescent psychiatry clinic in Omid Clinic and child and adolescent psychiatry subspecialty office in 2022–2023. They refer to the city of Babylon. The sample size was calculated based on the compare two means formula and the results of a previous similar study [11] using the Convenience Sampling method. The study sample included 120 children aged 8 to 12 years old diagnosed with attention deficit/hyperactivity disorder by a child and adolescent psychiatry specialist according to DSM-5 criteria, 60 of them with learning disorders and the remaining 60 without learning disorders.

Written informed consent was obtained from all parents after a thorough explanation of the study and its objectives. Child assent was also mandatory for participation. In cases where parents consented but the child did not, the child was not enrolled in the study. Also, all participants were assured that their personal information and names would be kept confidential. Data were stored securely and only accessible to authorized researchers. Results were analyzed anonymously, and no personally identifiable information was released.

### The inclusion and exclusion criteria of the study

In this study, patients over 8 years old and under 12 years old and diagnosed with attention deficit/hyperactivity disorder based on DSM-5 criteria, who had written consent and willingness to participate in the study, were included in the study. Patients with physical problems such as deafness or hearing loss, blindness or uncorrected visual impairment, etc., coexistence of other psychiatric diagnoses such as mental retardation, bipolar disorder, autism spectrum disorder, etc. ) and unwillingness and consent to participate were excluded from the study.

### Data collection method

Among the 8- to 12-year-old outpatients referred to the child and adolescent psychiatry clinic of Omid Babol Clinic, 120 children aged 8 to 12 years with attention deficit/hyperactivity disorder whose disease was diagnosed by a child and adolescent psychiatry subspecialist and based on criteria DSM-V diagnosis was confirmed, they were included in this study by following the inclusion and exclusion criteria. After introducing themselves to the child and his parents, the researchers explained about the subject of the research and the purpose of conducting it, and they were assured about the right to withdraw cooperation and the confidentiality of the details in the entire study process. Then, the questionnaires were provided to them and during the period of answering the questions, the researchers were available to them so that if they needed more explanation regarding the concepts and meanings of the words and other questions. In order to correct the effect of confounders and homogenize in two groups (ADHD group with learning disorder and another group without learning disorder), people were distributed equally in two groups in terms of age and gender as much as possible.

Among the tools used to collect information was the Colorado Learning Difficulties Questionnaire by Wilcott et al. (CLDQ). This questionnaire considers learning problems to consist of five basic factors of reading, arithmetic, social cognition, social anxiety and spatial functions that cause learning problems. The learning problems questionnaire consists of 20 items, which are completed by the students’ parents. Each statement is answered on a 5-point Likert scale from not at all [1] to always [5]: the higher the score, the more learning problems the child has. The validity of the learning problems questionnaire and its components have been checked by the creators of the questionnaire with internal consistency and retesting methods and acceptable values have been obtained. The internal consistency of the entire questionnaire and its subscales was estimated by calculating Cronbach’s alpha coefficient. Cronbach’s alpha coefficient for CLDQ and all its components is more than 0.70, so the internal consistency of the entire questionnaire and its subscales is confirmed. In order to check the validity of CLDQ through retesting, this questionnaire was administered on 20 parents of students with a time interval of two weeks, and using the Pearson correlation method, the test-retest reliability coefficient was obtained for the entire questionnaire and each of the components. Its values are greater than 0.70 and indicate the acceptability of the time similarity of CLDQ and its components.

In order to ensure the validity of the learning problems test, discriminant and construct validity have been investigated. The validity of the content in the Colorado Learning Difficulties Questionnaire has been checked and approved by the creators of the questionnaire. In this research, the accuracy and clarity of translation was confirmed through direct translation from English to Farsi and reverse translation from Farsi to English. In order to evaluate the discriminant validity of the Colorado Learning Difficulties Questionnaire, two normal and clinical groups were compared by performing the t-test. According to the data, it can be concluded that the average of the students of normal and special schools is significantly different in terms of the scores of the Colorado Learning Problems Questionnaire and its five subscales. Students of exceptional schools always have a higher average than students of normal schools. The obtained significant differences confirm the optimal discrimination power of the Colorado Learning Difficulties Questionnaire and its subscales. The construct validity of the Colorado learning problems scale has been investigated by two methods of calculating the correlation coefficient of the questionnaire with its subscales and exploratory and confirmatory factor analysis. There is a high correlation between the learning problems test and its five subscales. The intensity of the relationship between the Colorado Learning Difficulties Questionnaire and the reading subscales was 0.81, social cognition 0.78, social anxiety 0.76, spatial problems 0.70 and math 0.60. The significance of the mentioned relationships at a high level indicates that the Colorado learning problems scale has the desired construct validity [12].

The next instrument used is the five-scale self-esteem test of Pepe et al. (1989) for children. This test has five self-esteem scales (general, academic, physical, family, social scales) and a lie detector scale. Each scale has 10 separate questions and each question has 3 items (almost never, sometimes, always). In addition, obtaining a score of 2 in 4 items (or more) of the lie detector items indicates that the child has tried to respond in a sociable style and therefore the validity of the self-esteem scales may not be confirmed. After summing the points in each subscale; Very weak self-esteem, weak self-esteem, average self-esteem, good self-esteem, high self-esteem are categorized as follows. Cronbach’s alpha coefficient is based on the standard deviation of test questions. According to Cronbach’s alpha formula, the reliability coefficient of this test was calculated as α = 0.86, which compared to the method of halving the test, which is 0.87, the difference between the two methods is 0.01, which is very insignificant and negligible.

Following the Cronbach’s alpha method to find out the value of each question in the set of test questions, reliability was obtained after removing each question from the set of questions, which is known as the loop method. The reliability coefficient of the test is generally equal to 0.89.

In order to validate the test, methods of internal consistency and factor analysis were used. The internal consistency method is determined by calculating the correlation of each question with each test question with the corresponding scale, and with the whole test. The highest correlation of the whole test is with the general scale, which is *r* = 0.8346. In general, all scales show a significant correlation with the level of error probability of 0.001 [13, 14].

The next tool used is the quality-of-life questionnaire for 8–12-year-old children (PedsQL). The PedsQL questionnaire has different versions to measure the quality of life of children of different ages. The current version is designed to measure the quality of life of children aged 8 to 12 and has 23 questions. Questionnaire options are scored on a Likert scale. Finally, an overall score and four subscales are obtained for this questionnaire, which are: physical performance subscale, emotional performance subscale, social performance subscale, and academic performance subscale. Also, two general subscales of psychological health and physical health and a total score can be calculated for this questionnaire. The score obtained for each subscale will be between 0 and 100. A higher score means a higher quality of life and a lower score means a lower quality of life. In the study of Warney, Sid and Curtin (2001), the validity and reliability of this instrument was investigated in the United States. In this study, Cronbach’s alpha was calculated as 0.88 for children’s version and 0.90 for parents’ version. Mohammadian et al. (2013) investigated the validity and reliability of this questionnaire in children of Kashan city. In their study to check the content validity, the views of 9 faculty members of the universities were measured and the validity of the instrument was measured using the content validity index (CVI) method. The CVI calculated in this study was 0.84 for the whole instrument and 0.80 for the four subscales of physical performance, 0.86 emotional performance, 0.83 social performance and 0.88 school performance. Also, the results of the factor analysis confirmed the existence of the total score and four subscales. All items in the questionnaire had a factor loading above 0.40. Cronbach’s alpha was also calculated for the total score of 0.82 and for the subscales between 0.65 and 0.77 [15, 16].

### Data analysis method

SPSS version 22 software will be used for data analysis. Descriptive statistics will be presented using mean and standard deviation (for quantitative data) and frequency and proportion (for qualitative data). Chi-square, independent t-test and ANOVA tests were be used to check the desired relationships. *P* > 0.05 is considered significant.

## Results

### Demographic information

120 children with attention deficit/hyperactivity disorder were included in the present study, including 62 (51.7%) boys. The average age of these children is 9.7 ± 1.42 (minimum = 8 and maximum = 12) years. Among 120 mothers, 87 (72.5%) were housewives and 33 (27.5%) were employed. Among 120 fathers, 49 (40.8%) were employed and 71 (59.2%) were self-employed. The education of 33 (27.5%) mothers is below diploma, 56 (46.7%) have diploma and 31 (25.8%) have above diploma. The education of 40 people (33.3%) of fathers is below diploma, 50 people (41.7%) have diploma and 30 people (25%) have above diploma.

### Colorado Learning Difficulties Questionnaire (CLDQ)

The results showed that the average score obtained from the questionnaires was 47.37 ± 15.43. The average scores in reading area were 14.9 ± 7.29, social cognition area was 8.54 ± 3.83, social anxiety area was 6.43 ± 2.89, spatial problems area was 9.48 ± 3.82, and math problems area was 8.21 ± 3.45. Among the 60 children with learning disabilities, there are 26 children who have disabilities in all five areas. 12 children also have learning difficulties in only one specific area: three in reading, three in social cognition, four in spatial, and two in math. The remaining 22 people have learning disabilities in two to four areas (Table 1).

**Table 1:** The frequency of people with SLD according to the subscales of the CLDQ questionnaire

Variable	Frequency(n)	Percentage in the group with SLD	Percentage in the total group
**Loneliness of the reading domain**	3	5	2.5
**Loneliness disorder of social cognition**	3	5	2.5
**Loneliness disorder in the area of social anxiety**	0	0	0
**Loneliness disorder of the spatial domain**	4	6.7	3.33
**Loneliness disorder in the field of mathematics**	2	3.3	1.66
**Disruption in all areas**	26	43.3	21
**Other (disruption in at least two areas and at most four areas)**	22	36.7	18.33

### Self-esteem test of Pepe

The average score of this questionnaire was calculated as 85.73 ± 11.96. The distribution of the fragments of its scales is given in Table 2.

**Table 2:** Average distribution of the subscales of Pep et al.‘s self-esteem questionnaire

Variable	Minimum	Maximum	Mean	Standard deviation
**Total self-esteem**	38	110	85.73	11.96
**General**	5	20	17.38	2.87
**educational**	5	20	13.5	3.77
**physical**	1	20	15.65	3.02
**family**	4	20	15.31	3.1
**social**	6	19	14.29	2.81

### Quality of life questionnaire

The data analysis shows that the average score of the questionnaire filled by children was 74.07 ± 12.7 and that of parents was 68.3 ± 14.72. (Table 3)

**Table 3:** Average distribution of subscales of the PedsQL questionnaire in two child and parent forms

Variables	Minimum		Maximum		Mean		Standard deviation	
	child	parent	child	parent	child	parent	child	parent
**Total quality**	40.21	29.34	100	97.82	74.07	68.30	12.70	14.72
**Physical performance**	28.12	18.75	96.87	100	80.11	78.77	15.10	21.26
**Emotional functioning**	15	10	100	100	69.04	58.95	18.90	22.43
**Social Performance**	15	25	100	100	72.54	71.12	20.45	19.87
**Academic Performance**	25	20	100	100	64.45	60.54	16.57	15.95
**Psychological health**	13.60	11.66	100	96.66	67.87	62.57	15.27	15.41
**Physical health**	28.12	18.75	96.87	100	80.11	78.77	15.10	21.26

The results show that the average total self-esteem and its subscales in the group of children with ADHD without learning disorders is higher than the group with learning disorders. However, this issue is an exception in the family self-esteem subscale (ADHD + LD: 15.46 and ADHD: 15.16) which was not statistically significant(*P*.value = 0.599). The result has shown that only in the academic subscale, the difference between the two groups with and without learning disorders is significant (ADHD + LD: 12.58 and ADHD: 14.41) (*P*-value = 0.007). More details are given in Table 4.During statistical investigations to compare the average quality of life between the two studied groups, it was seen that in the parent form of quality of life, the average quality of life and its subscales in the group of children with ADHD without learning disorders is higher than in the group with co-occurring disorders. All these differences are significant (P-value < 0.05) except for the difference in the subscale of emotional performance (*P*-value = 0.064).

**Table 4:** Comparison of the average self-esteem of two ADHD groups with and without the co-occurrence of learning disorders

Variable	Groups Mean ± SD		*P*-value
	ADHD	ADHD + LD	
**Self-esteem**	87.15 ± 12.62	84.31 ± 11.18	0.196
**General**	17.50 ± 2.75	17.26 ± 3.00	0.658
**educational**	14.41 ± 3.69	12.58 ± 3.64	**0.007**
**physical**	15.71 ± 3.03	15.58 ± 3.03	0.810
**family**	15.16 ± 3.21	15.46 ± 3.00	0.599
**social**	14.40 ± 2.80	14.18 ± 2.84	0.675

In the child form of quality of life, the average quality of life and its subscales in the group of children without learning disabilities is higher than in the group with learning disabilities, except for the subscale of emotional functioning(ADHD:68.16 vs. ADHD + LD:69.91). The existing differences are significant only in terms of physical performance(*P*.value = 0.017), physical health(*P*.value = 0.017) and academic performance(*P*.value = 0.022) (Table 5).

**Table 5:** Comparing the average quality of life of two ADHD groups with and without co-occurring learning disorder

Variable	parent form			Child form		
	Groups Mean ± SD		*P*-value	Groups Mean ± SD		*P*-value
	ADHD	ADHD + LD		ADHD	ADHD + LD	
**Quality of Life**	73.72 ± 13.12	62.88 ± 14.32	**≤ 0.001**	75.88 ± 12.27	72.25 ± 12.96	0.119
**Physical performance**	85.00 ± 19.36	72.54 ± 21.40	**0.001**	84.37 ± 11.66	76.52 ± 16.78	**0.017**
**Emotional functioning**	62.75 ± 22.65	55.16 ± 21.74	0.064	68.16 ± 19.78	69.91 ± 18.09	0.614
**Social Performance**	78.75 ± 17.65	63.50 ± 19.16	**≤ 0.001**	74.91 ± 19.18	70.16 ± 21.54	0.205
**Academic Performance**	66.41 ± 15.37	54.66 ± 14.37	**≤ 0.001**	67.91 ± 16.98	61.00 ± 15.53	**0.022**
**Psychological health**	67.71 ± 15.28	57.44 ± 13.84	**≤ 0.001**	69.05 ± 16.28	66.69 ± 14.23	0.399
**Physical health**	85.00 ± 19.36	72.54 ± 21.40	**0.001**	84.37 ± 11.66	76.52 ± 16.78	**0.017**

## Discussion

ADHD is strongly associated with a number of psychiatric and physical disorders, including learning disabilities [17]. When ADHD is co-occurring with learning disabilities, treatment becomes more complicated and children are severely affected. A better understanding of the high prevalence of ADHD comorbidities is necessary to optimize the treatment of this disease and prevent some of the negative outcomes associated with comorbid ADHD [17]. The relationship between self-esteem, quality of life (QoL) and attention-deficit/hyperactivity disorder (ADHD) in children is complex and multifaceted. The association of learning disorder with ADHD may make this association even more complicated. The results of this study showed that learning disorders in 5 areas are seen in more than 40% of ADHD children. Total self-esteem and its subscales were lower in children with ADHD with learning disorder, except for the subscale of family self-esteem and quality of life, it was also lower in children with ADHD with learning disorder.

In the present study, out of 120 children with attention deficit/hyperactivity disorder, 51.7% were boys and 48.3% were girls. In line with this finding, there are other studies, such as in the study of Jelinkova et al. (2022) where 63.46% were boys and 36.53% were girls [18], as well as Sorrenti et al. (2019) where 61.9% were boys and 38.09% were girls [19].

The results of the study showed that children with ADHD and learning disorders have lower academic self-esteem compared to the group without learning disorders, and the more these learning disorders increase, the more the children’s academic self-esteem decreases. In line with this finding, a study was conducted by Chahardoli et al. They found that students with specific learning disabilities face many academic problems and emotional frustrations. These students have a negative self-concept due to being prone to academic failure, being labeled as a disorder and separating them from the normal education process that many of them experience. These children receive a lot of negative feedback at school and have less successful experiences in academic areas. Compared to their normal classmates, students with specific learning disabilities often get lower grades and are usually not given enough attention by their teachers and are rejected by their friends and peers; Therefore, this finding can be explained in this way that the repeated experience of the mentioned problems and their internalization creates a series of negative, resistant and recurring thoughts, around the axis that “I am a disabled person” in these students and reduces their ability to Using effective problem solving strategies reduces [20]. Another study focused on self-esteem and clinical characteristics in children with ADHD and social anxiety disorder (SAD) and found that children with ADHD and SAD had lower self-esteem compared to children without SAD [21]. This shows that the association of ADHD with other mental illnesses can have a greater impact on self-esteem.

The results of this study showed that no child from the group with learning disorders is in the high self-esteem group and most of them are in the average range of overall self-esteem. If children without learning disorders have achieved high self-esteem. In line with this finding, Salik et al. investigated psycho-social problems arising from special learning disorders during adolescence. They concluded that children diagnosed with SLD have both internalizing problems, such as low self-esteem and social disadvantage, and externalizing concerns, such as difficulty making friends, socializing, and engaging in delinquent activities. Therefore, to help children with SLD to overcome related problems, it is recommended to provide comprehensive education and implement therapeutic interventions [22]. Also, parental influence and interventions can play an important role in the self-esteem and quality of life of children with ADHD. Parenting styles are associated with self-esteem levels in children with ADHD, and authoritarian parenting is associated with low implicit self-esteem [23].

Quality-of-life is increasingly valued as a key element in understanding the impact of health problems on children, particularly when it comes to mental health issues [24, 25]. The results obtained from the parent form of the quality-of-life questionnaire showed that children with ADHD and learning disorders have a lower quality of life in the areas of physical, social, academic, physical health and psychological health compared to the group without learning disorders. The child form of the questionnaire has shown this significant difference only in the physical and academic fields. In the following, it was also seen that the higher the amount of learning disorders, the lower the quality of life of children in these areas. A systematic review and meta-analysis examining the association between children’s health-related quality of life and ADHD found that ADHD was associated with significantly poorer children’s quality-of-life. Interestingly, parents rated their children’s quality-of-life lower than the children themselves [26]. A previous study stated that children with ADHD are prone to frequent and severe functional physical symptoms (such as stomach pain, fatigue, and headache) that lead to poor quality of life with more emotional and behavioral problems than children without ADHD [27]. Also, in another study [28], researchers reported that children’s low quality of life can be worsened by ADHD symptoms, concurrent health conditions (such as autism spectrum disorder), problems with peers, behavior and coordination, as well as family issues such as illness and parental behaviors (such as e.g., depression/stress, maternal smoking) and family composition (e.g., divorce, living apart). Understanding these factors contributing to poor quality of life is important to evaluate and improve treatment options for ADHD in children. In line with the findings, Commodari et al. investigated interpersonal compatibility, general self-efficacy and metacognitive skills in first and second high school students with and without special learning disorders in an observational and prospective study. Their subjects completed a series of standardized tests to evaluate social and interpersonal skills (disapproval, impulsivity, narcissism, social preoccupation, and stress in social situations), general self-efficacy and metacognitive skills. The results showed that students with SLD have lower interpersonal compatibility than non-disabled students. Also, people with special learning disorders are more impulsive and have many problems in managing social situations. They also reported lower levels of self-efficacy [29]. Also, in another study, they investigated the international research conducted on the psychosocial characteristics of students with high potential but with SLD. The results showed that all these students have a common psycho-social profile, which actually arises from the combination of learning disorder and their inner talent: frequent and long-term academic failure, feelings of disappointment due to unbalanced skills, unfavorable relationships with peers, and the feeling of not belonging, all of which can lead to behavioral disorders, anxiety, depression and other problems. Therefore, it is important to support these students not only to meet their academic needs, but also their emotional and social needs in order to prevent behavioral and emotional disorders [30]. They also conducted a study to investigate the relationship between important clinical factors, namely the severity of ADHD symptoms, its association with other disorders and the level of motor skills with the quality of life of these people. In general, it was concluded that the coexistence of other disorders with ADHD, due to the increase in the severity of ADHD symptoms and further reduction of motor skills, can play an important role in predicting the quality of life of these people [31]. Therefore, it is important to confirm the wider impact of ADHD on the daily functioning of children and adolescents so that adequate support can be provided for children with ADHD. Considering that the school environment is strongly affected by ADHD, as shown in the previous study [26], educational support for children with ADHD is essential. Improving children’s health-related quality of life is increasingly recognized as a key goal of ADHD treatment alongside clinical treatment outcomes and improved functional outcomes [25], thus, routine inclusion of general HRQoL measures in health interventions for children. Encouraged by ADHD. Future research should also incorporate parent and child perspectives on measures of children’s health-related quality of life, as both appear to be important in understanding the broad impact of ADHD on a child’s daily life functioning/well-being.

### Limitations of the study

Due to the young age of the children and their underlying disorder, there was a lack of proper cooperation in some cases.

Despite the moderator’s attention to having relatively similar samples, there were still many factors such as family problems, economic problems, separation of parents, loss of loved ones, etc., which could affect children’s self-esteem and quality of life.

## Conclusion

Children with ADHD and learning disabilities experience lower self-esteem and quality of life compared to those with ADHD alone. This highlights the importance of comprehensive assessments to identify co-occurring disorders. Early diagnosis can lead to interventions that address both ADHD and learning disabilities, potentially improving children’s self-esteem and quality of life across physical, social, academic, and psychological domains. As mentioned, ADHD leads to significant impairment in academic, occupational, familiarity, and social performance, and its consequences can also exist in adulthood [32]. For researchers, these findings suggest a need for further exploration of the discrepancies in quality-of-life reports and the development of effective interventions for this population. For teachers, understanding these challenges can inform strategies for supporting self-esteem and addressing potential difficulties in various domains within the classroom environment and for psychotherapists, considering the co-occurrence of ADHD and learning disabilities is crucial for developing comprehensive treatment plans that address both conditions and improve overall well-being.

## Data Availability

The data used during the current study are available from the corresponding author on reasonable request.
